# Sensitivity and Specificity of a Novel Classifier for the Early Diagnosis of Dengue

**DOI:** 10.1371/journal.pntd.0003638

**Published:** 2015-04-02

**Authors:** Nguyen Minh Tuan, Ho Thi Nhan, Nguyen Van Vinh Chau, Nguyen Thanh Hung, Ha Manh Tuan, Ta Van Tram, Nguyen Le Da Ha, Phan Loi, Han Khoi Quang, Duong Thi Hue Kien, Sonya Hubbard, Tran Nguyen Bich Chau, Bridget Wills, Marcel Wolbers, Cameron P. Simmons

**Affiliations:** 1 Children’s Hospital No. 1, Ho Chi Minh City, Vietnam; 2 Oxford University Clinical Research Unit, Hospital for Tropical Diseases, Ho Chi Minh City, Vietnam; 3 Hospital for Tropical Diseases, Ho Chi Minh City, Vietnam; 4 Children’s Hospital No. 2, Ho Chi Minh City, Vietnam; 5 Tien Giang Provincial Hospital, My Tho, Tien Giang Province, Vietnam; 6 Dong Nai Children’s Hospital, Bien Hoa, Dong Nai Province, Vietnam; 7 Long An Provincial Hospital, Tan An, Long An Province, Vietnam; 8 Binh Duong Provincial Hospital, Thu Dau Mot, Binh Duong Province, Vietnam; 9 Department of Microbiology and Immunology, University of Melbourne, Parkville, Victoria, Australia; 10 Centre for Tropical Medicine, Nuffield Department of Medicine, University of Oxford, Oxford, United Kingdom; 11 Nossal Institute of Global Health, University of Melbourne, Parkville, Victoria, Australia; Pediatric Dengue Vaccine Initiative, UNITED STATES

## Abstract

**Background:**

Dengue is the commonest arboviral disease of humans. An early and accurate diagnosis of dengue can support clinical management, surveillance and disease control and is central to achieving the World Health Organisation target of a 50% reduction in dengue case mortality by 2020.

**Methods:**

5729 children with fever of <72hrs duration were enrolled into this multicenter prospective study in southern Vietnam between 2010-2012. A composite of gold standard diagnostic tests identified 1692 dengue cases. Using statistical methods, a novel Early Dengue Classifier (EDC) was developed that used patient age, white blood cell count and platelet count to discriminate dengue cases from non-dengue cases.

**Results:**

The EDC had a sensitivity of 74.8% (95%CI: 73.0-76.8%) and specificity of 76.3% (95%CI: 75.2-77.6%) for the diagnosis of dengue. As an adjunctive test alongside NS1 rapid testing, sensitivity of the composite test was 91.6% (95%CI: 90.4-92.9%).

**Conclusions:**

We demonstrate that the early diagnosis of dengue can be enhanced beyond the current standard of care using a simple evidence-based algorithm. The results should support patient management and clinical trials of specific therapies.

## Author Summary

Dengue is a very common acute infectious disease in the tropical world. Health care professionals are able to better care for dengue patients if they can make an early diagnosis and make a plan for case management. This current study investigated fever in 5729 children in Vietnam with 3 days or less of fever and identified 1692 dengue cases using advanced, gold standard methods. We systematically collected a range of medical and laboratory findings on each patient when they entered the study and used statistical tools to determine if these medical and laboratory findings could enable early diagnosis, independent of sophisticated, gold-standard laboratory tests. Our results, called the Early Dengue Classifier, had performance characteristics suggesting it could improve the diagnostic proficiency of health care professionals. However the performance of the Early Dengue Classifier is not perfect and likely will not change the practice of experienced doctors in dengue endemic settings. Our study highlights the need for 2^nd^ generation, easy-to-use rapid diagnostic tests that can accurately diagnose dengue in the first few days of fever.

## Introduction

Dengue is an acute, systemic viral infection and a public health problem in the tropical world [1]. The etiological agents of dengue are any of the four dengue viruses (DENV-1-4). In endemic countries it is common for all four DENV serotypes to co-circulate. Late-stage trials of a dengue vaccine with intermediate efficacy have recently been reported, offering hope of a public health intervention [2, 3].

The World Health Organisation (WHO) has a stated goal of reducing global dengue mortality by 50% by 2020 [1]. Improvements in case diagnosis and management will be central to achieving this aim. Significant loss of intravascular plasma volume leading to hypovolemic shock (dengue shock syndrome (DSS)), usually between the 4^th^-6^th^ day of illness, is the commonest life-threatening complication of dengue [1, 4]. It’s widely held that the case-incidence of DSS can be reduced via careful monitoring and the judicious use of parenteral fluids to maintain an adequate intravascular volume [1]. Ideally, this case management approach is enabled because the attending physician had made an early diagnosis and thus alerted clinicians, nurses and family caregivers to the signs and symptoms suggestive of clinical worsening. Additional benefits of an early diagnosis include support to community level public health interventions and improvements in the sensitivity of case surveillance systems and disease burden estimates. Furthermore, it is likely that the therapeutic window of opportunity for a dengue antiviral drug lies in the first 48–72 hours of illness [5]. Thus, programmatic use of therapeutic interventions in the future will likely go hand in hand with strategies for early diagnosis.

Yet there are numerous challenges for busy primary care clinicians in making a diagnosis of dengue in the first few days of illness. Rapid lateral flow tests, based on the detection of the viral NS1 antigen, are available in some settings and can provide a confirmatory diagnosis [6–8]. The diagnostic performance of the WHO dengue case definition, which relies on non-specific signs and symptoms that overlap with other infectious diseases, is unknown in the first few days of illness [1]. Potts *et al* concluded that more prospective studies were needed to construct a valid and generalizable algorithm to guide the differential diagnosis of dengue in endemic countries [9]. To this end, several prospective studies have described the creation of classifiers for the diagnosis of dengue [10–12]. However none of these studies have exclusively focused on pediatric fever cases presenting to primary care facilities with short illness histories, a very common scenario in dengue endemic settings. Against this backdrop, the purpose of the current study was to prospectively derive a dengue diagnostic algorithm from routinely collected clinical and laboratory findings in pediatric patients with <72 hours of illness history and compare this approach against the diagnostic performance of a leading NS1 rapid test (BioRad NS1 Ag STRIP) in the same patients. The results provide pragmatic methods to enhance the early diagnosis of dengue in primary care settings.

## Materials and Methods

### Human research ethics

The study protocol was approved by the Hospital for Tropical Diseases scientific and ethical committee and the Oxford University Tropical Research Ethical Committee (OXTREC 35–10). The accompanying parent/guardian of each child provided written informed consent.

### Patient enrolment

Recruitment occurred in the public sector outpatient departments of Children’s Hospital No. 1 (HCMC), Children’s Hospital No. 2 (HCMC), The Hospital for Tropical Diseases (HCMC), Tien Giang Provincial Hospital, Dong Nai Children’s Hospital, Binh Duong Provincial Hospital and Long An Provincial Hospital. These outpatient departments function as primary care providers to their local communities. A patient presenting to one of the study sites was eligible for enrolment if they met the following inclusion criteria—a) fever at presentation (or history of fever) and less than 72 hours of symptom history, b) in the attending physicians opinion dengue was a possible diagnosis, c) 1–15 years of age inclusive, d) accompanying family member or guardian had a mobile phone and e) written informed consent for the child to participate was provided by the parent/guardian. Patients were excluded if- a) the attending physician believed they were unlikely to be able to attend follow-up or b) the attending physician believed another (non-dengue) diagnosis was more likely. Patient enrolment occurred consecutively during normal clinical hours on weekdays without restriction. All patients were enrolled into the study before the attending physician received the results of any routine laboratory tests.

### Clinical and laboratory investigations on the day of enrolment

At the time of enrolment, information on the patient’s age, sex, illness history, presenting signs and symptoms were recorded in a case report form. The definitions used to support standardized data capture are shown in S1 Table. Blood samples were drawn for routine hematology, biochemistry and NS1 rapid test. All NS1 rapid tests (NS1 Ag STRIP, BioRad) were performed on the same day of patient enrolment by one of two trained laboratory technicians at the Hospital for Tropical Diseases. Routine hematology results, but not biochemistry or NS1 rapid test results, were made available to the attending physician, who decided on the management approach, i.e. hospitalization or ambulatory follow-up.

### Patient follow-up

A 2^nd^ blood sample for the purposes of serology was collected around the time of defervescence from all patients that were hospitalized anytime during their acute illness. If the patient was managed solely on an ambulatory basis for the duration of their illness, then a 2^nd^ early convalescence blood sample for the purposes of serology was collected only from a randomly selected 10% of this patient population. The randomization code to select ambulatory cases for follow-up was generated by software. All clinical and laboratory data were stored in an ICH-GCP compliant, clinical data management platform called “CLIRES”. Demographic and clinical data were double entered. Electronic data files containing hematological results were uploaded directly to CLIRES. Independent study monitoring was performed by the Clinical Trials Unit of the Oxford University Clinical Research Unit which examined adherence to the trial procedures, data collection and recording and compliance with ICH-GCP.

### Dengue diagnostics and case definitions

The gold standard diagnostic result was a composite derived from three tests; RT-PCR, IgM serology and NS1 detection by ELISA. First, all enrolment plasma samples were tested with a validated, quantitative RT-PCR assay to detect DENV RNA [13]. Next, any enrolment plasma samples that were negative in the RT-PCR assay were tested using the Platelia Dengue NS1 Ag ELISA assay (BioRad) and scored according to the manufacturer's instructions. Samples with equivalent results were repeated and if still equivocal they were scored as negative. Next, IgM ELISA serology (Panbio, Brisbane, Australia) was performed according to the manufacturer's instructions for patients who had paired plasma samples (enrolment and early convalescence) and who were negative in both the DENV RT-PCR assay and Platelia Dengue NS1 ELISA. Any patient who was—a) DENV RT-PCR positive, b) NS1 ELISA positive, or c) had DENV IgM seroconversion in paired plasma samples, was classified as a laboratory-confirmed dengue case. IgM seroconversion was defined as a change in the MAC ELISA test result from negative to positive in paired plasma samples with the 2nd sample collected 6 or more days after illness onset and >2 days after the 1st sample.

Any patient who was DENV RT-PCR negative, NS1 ELISA negative and did not IgM seroconvert in paired plasma samples was classified as “not dengue”. Any patient who was DENV RT-PCR negative and NS1 ELISA negative at the time of enrolment, but did not have paired samples available for serology, was classified as a “presumptive not-dengue” case. For analysis, data from “not dengue” and “presumptive not-dengue” cases were pooled.

### Enrichment of NS1

Plasma samples were enriched for proteins with molecular weight >100kDa using Amicon filtration units (Millipore). Briefly, 200μl of plasma was concentrated to ~30μl and then tested in the Platelia NS1 ELISA. All concentrated samples were tested in parallel with an aliquot of the original plasma samples and the filtrate (containing proteins with molecular weight <100kDa).

### Statistical methods

Logistic regression was used for the development of the diagnostic algorithm. A detailed assessment of the model assumptions of linearity and additivity was performed (S1 Text). All pre-defined candidate predictors listed in S1 Table and significant interaction terms were included in the full model. The model was then simplified using step-wise backwards selection using Akaike’s Information Criterion (AIC) and stability selection [14]. Alternative statistical models such as classification and regression trees (CART) and random forests (RF) were also investigated in order to find an optimal diagnostic algorithm [15, 16]. The performance of the model was assessed with respect to discrimination (receiver operating characteristic curves (ROCs) and area under the ROC curve (AUC)), calibration (calibration plots and calibration intercepts and slopes), and standard accuracy criteria of binary diagnostic tests (sensitivity, specificity, negative and positive predictive values). We selected the cut-off point to classify a patient as dengue positive at a predicted risk of dengue of ≥33.3%, corresponding to assuming that the “cost” of missing a true dengue patient is twice as large as the cost of a false-positive [17]. To avoid over-optimistic estimates of model accuracy and performance due to model derivation and evaluation on the same dataset, all accuracy measures were corrected for optimism by validation. Validation was performed for the whole model development process including variable selection. Two validation schemes were employed to mimic external validation:

- “leave-one-site-out cross-validation”, i.e. repeatedly developing the algorithm on all but one site and validation on the left-out site
- Temporal validation with patients recruited before 15 June 2012 as the training set and patients recruited thereafter as the evaluation set [18].

The final logistic model was also presented as a nomogram for direct clinical use. All statistical analyses were performed using the statistical software R v3.1.1 (R foundation for statistical computing, Vienna, Austria) and its companion packages c060 version 0.2–3 (for stability selection), randomForest version 4.6–7 (for random forest) and rpart version 4.1–8 (for CART).

## Results

### Study population

5729 children with fever of less than 72 hours were enrolled at one of the seven clinical study sites in southern Vietnam between October 2010 and December 2012. A summary of the patient screening, enrolment and diagnostic outcomes is shown in S1 Fig A total of 5707 patients were included in the analyses. 1692 (29.6%) participants had laboratory-confirmed dengue. The baseline characteristics of the dengue and non-dengue cases are shown in Table 1. Notably, dengue cases were older than non-dengue cases. All four DENV serotypes were detected; DENV-1 was the commonest serotype, followed by DENV-4, -2 and -3.

**Table 1:** Baseline characteristics of study participants.

	Laboratory-confirmed dengue (n = 1692)	Non-Dengue (n = 4015)
**Demographic characteristics**		
Age (years)	9 (6–11)	5 (3–8)
Sex-Male (n, %)	945 (55.9%)	2249 (56.0%)
BMI (kg/(m)^2^)	16.4 (14.6–18.9)	15.6 (14.1–17.6)
**History and clinical characteristics**		
Day of illness		
1	361 (21.3%)	1232 (30.7%)
2	692 (40.9%)	1732 (43.1%)
3	639 (37.8%)	1051 (26.2%)
Temperature (°C)	38.5 (38–39)	38.4 (37.8–39.0)
Vomiting (n, %)	737 (43.6%)	1442 (35.9%)
Abdominal pain (n, %)	351 (20.7%)	702 (17.5%)
Skin bleeding (n, %)	243 (14.4%)	135 (3.4%)
Mucosal bleeding (n, %)	113 (6.7%)	99 (2.5%)
Flush (n, %)	399 (23.6%)	532 (13.3%)
Hepatomegaly (n, %)	6 (0.4%)	5 (0.1%)
Rash (n, %)	62 (3.7%)	79 (2.0%)
Conjunctival injection (n, %)	343 (20.3%)	385 (9.6%)
Laboratory results*		
WBC (10^3^/mm^3^)	4.74 (3.50–6.80)	8.90 (6.36–12.40)
PLT (10^3^/mm^3^)	180 (141–227)	242 (200–292)
HCT (%)	38.6 (36.6–40.7)	37.4 (35.3–39.6)
ALB (g/L)	43.7 (41.7–45.6)	43.9 (42.0–45.7)
AST (U/l)	51 (40–67)	42 (35–49)
CK (U/l)	105 (82–140)	100 (76–131)

* All laboratory results were acquired on the day of enrolment.

Values are presented as median and interquartile range for continuous variables or frequency and percentage for categorical variables.

BMI: body mass index; WBC: white blood cell count; PLT: platelet count; HCT: hematocrit; ALB: albumin; AST: aspartate aminotransferase; CK: creatine kinas

### Diagnostic accuracy of the NS1 Ag Strip test and association with viremia

Enrolment plasma samples (n = 5707) were tested for the presence of NS1 by NS1 Ag Strip test in a blinded, real-time fashion. Against the composite gold-standard reference diagnostic result, the NS1 Ag Strip test had a sensitivity of 70.4% (95%CI: 68.2–72.6%), specificity of 99.2% (95%CI: 98.9–99.5%), positive predictive value (PPV) of 97.4% (95%CI: 96.3–98.2%), and negative predictive value (NPV) of 88.9% (95%CI: 87.9–89.8%) for the diagnosis of dengue (Table 2). There was a striking difference in diagnostic performance by serotype, with NS1 detection being less sensitive in DENV-2 infections irrespective of the serological response (primary vs secondary)(S2 Table). The detection of NS1 was strongly associated with the concentration of DENV RNA in the same plasma sample; the odds of NS1 detection increased by 1.8 (95%CI: 1.6–1.9) for each 10-fold higher DENV RNA concentration (Table 2). These data define the strengths and weaknesses of NS1 rapid testing; it is highly specific but is compromised by suboptimal sensitivity, especially for DENV-2 cases.

**Table 2:** Diagnostic performance of NS1 rapid test in enrolment plasma samples and odds of NS1 detection in relation to plasma viremia.

	Laboratory-confirmed dengue cases	Non-dengue cases	Total	
NS1 rapid test positive	1192	32	1224	PPV % = 97.4% (96.3–98.2%)
NS1 rapid test negative	500	3983	4483	NPV % = 88.9% (87.9–89.8%)
Total	1692	4015	5707	
	Median (IQR) plasma viral RNA concentration (log10copies/ml)^a^	OR (95%CI)	Sensitivity % (95%CI)	Specificity % (95%CI)
All serotypes (n = 1692)	7.3 (6.2–8.3)	1.8 (1.6–1.9)	70.4% (68.2–72.6%)	99.2% (98.9–99.5%)
DENV-1 (n = 629)	7.9 (6.6–8.7)	2.0 (1.8–2.3)	80.3 (77.0–83.3%)	-
DENV-2 (n = 399)	7.0 (6.0–7.9)	1.8 (1.5–2.1)	46.4 (41.4–51.4%)	-
DENV-3 (n = 154)	7.5 (6.4–8.6)	1.4 (1.1–1.9)	85.1 (78.4–90.3%)	-
DENV-4 (n = 433)	6.9 (6.0–7.7)	1.5 (1.3–1.8)	75.8 (71.3–79.7%)	-

^a^ Viremia measurement in the enrolment plasma sample (the same sample was also used for NS1 testing).

PPV: positive predictive value; NPV: negative predictive value; DENV: dengue virus; OR: odds ratios for detecting NS1 for each 10-fold higher DENV RNA concentration. There were 77 dengue cases where the infecting serotype was unknown.

### Manipulation of plasma specimens to improve NS1 detection

Volume enrichment of the plasma molecular weight fraction containing multimeric NS1 (>100,000kDa) was performed on plasma samples from 21 viremic dengue cases enrolled in this study. However, despite 5-10-fold concentration of plasma, this processing failed to materially improve the diagnostic yield, with only 1 of 11 samples changing their status from negative (original sample) to positive (concentrated sample) in the Platelia NS1 ELISA (S3 Table).

### The Early Dengue Classifier; A diagnostic rule based on clinical and simple laboratory features

**Fig 1:**
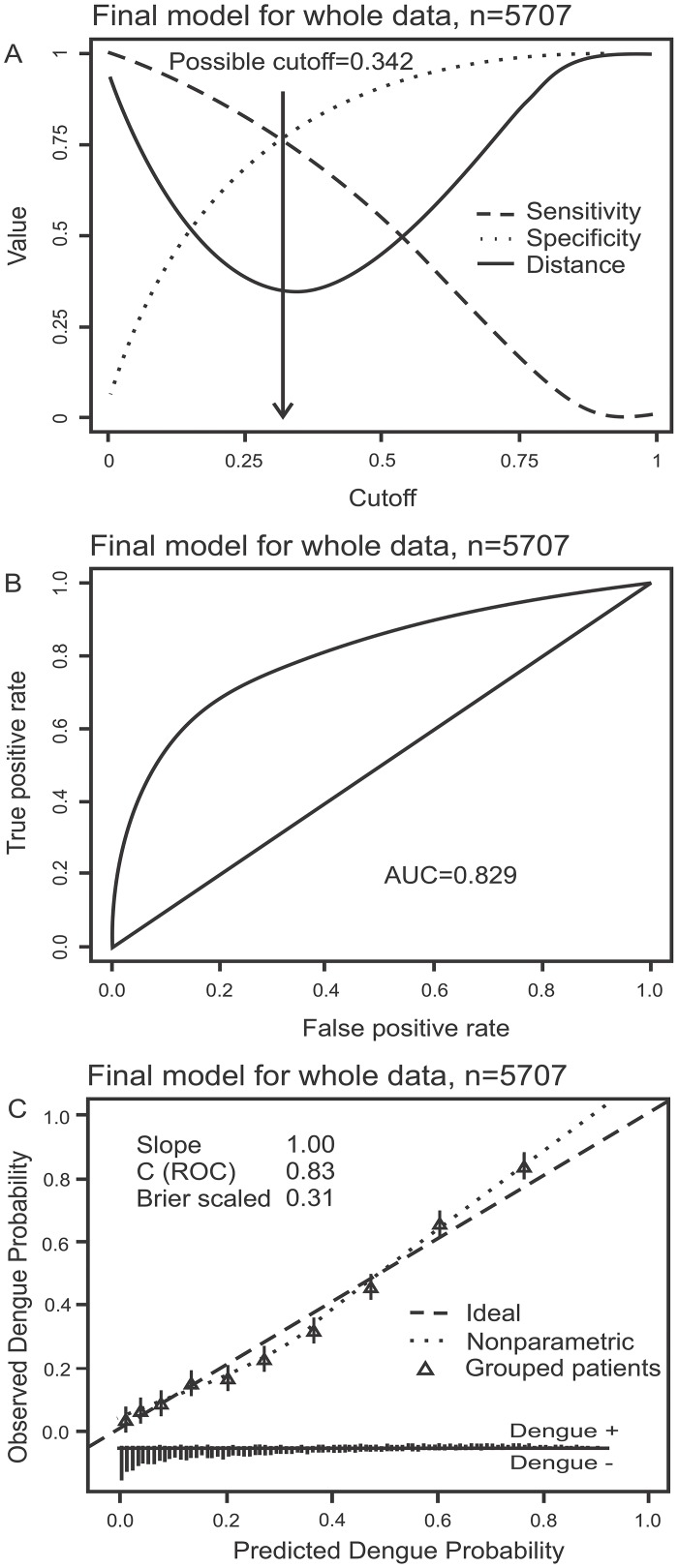
Performance of the Early Dengue Classifier (EDC) in all subjects. Figure A displays possible sensitivity/specificity trade-offs for different cut-off values and the distance from the corresponding points on the ROC curve to the upper left corner (perfect model). Figure B displays the receiver operating characteristic (ROC) curve. Figure C is a calibration plot. It displays a scatterplot-smoother of predicted versus observed risks (dotted line), predicted versus observed risks for ten patient strata of equal size grouped according to predicted risks (triangles) and the ideal identity line (dashed line). The rugs at the bottom of the graphs characterize the distribution of predicted risks in true dengue and non-dengue cases, respectively.

Multivariate logistic regression analyses of clinical, demographic and laboratory data from 5707 patients were performed to generate a practical dengue diagnostic classifier that could replace or augment NS1-based diagnosis in the first 72 hours of illness. The most parsimonious model, derived from stability selection, used the patient’s age, white cell count and platelet count at the time of enrolment to classify dengue from non-dengue cases (Table 3). Alternative approaches to feature selection yielded models with only slightly higher performance but relied on many more (more than ten) variables (S4 Table). The most parsimonious model, herein called the Early Dengue Classifier (EDC), had a sensitivity of 74.8% (95%CI: 73.0–76.8%), specificity of 76.3% (95%CI: 75.2–77.6%), positive predictive value of 57.1% (95%CI: 56.2–59.0%), and negative predictive value of 87.8% (95%CI: 86.8–88.5%) for correctly classifying dengue cases in the entire dataset at the pre-defined cut-off of 33.3%. Of note, this pre-defined cut-off reflecting clinical priorities was very close to the cut-off corresponding to the point on the ROC curve closest to the upper left corner (perfect model), which was 34.2% (Fig 1A). The area under the ROC curve (AUC) was 0.829 (Fig 1B) and the predicted risk of dengue agreed well with the observed risk (Fig 1C). The EDC had sensitivity of 72.9% (95% CI: 69.6–76.6%) for DENV1, 74.7% (95%CI: 71.0–79.7%) for DENV2, 68.4% (95%CI: 59.2–74.5%) for DENV3 and 78.2% (95%CI: 75.5–83.3%) for DENV4 infection. The overall performance characteristics of the EDC under temporal, leave-one-site-out validation or seasonality (rainy versus dry season), are summarized in S5 Table. These results suggest that, in settings where NS1 rapid tests are not routinely available, the EDC could assist primary care physicians in dengue diagnosis.

**Table 3:** Univariate and multivariate analysis of candidate predictors of laboratory-confirmed dengue.

	Univariate analysis			Multivariate analysis					
				Full model with all candidate predictors			Final model based on stability selection		
	OR	95% CI	p	OR	95% CI	P	OR	95% CI	p
**Demographic characteristics**									
Age (by + 1 year)	1.24	1.22–1.26	<0.001	1.21	1.18–1.24	<0.001	1.15	1.13–1.17	<0.001
Sex: Male	0.99	0.89–1.11	0.909	0.94	0.81–1.09	0.432	-	-	-
BMI (by +1)	1.09	1.07–1.11	<0.001	1.06	1.04–1.09	<0.001	-	-	-
**History and clinical characteristics**									
Day of illness (+1 day)	1.45	1.34–1.56	<0.001	0.78	0.70–0.87	<0.001	-	-	-
Temperature (by +1°C)	1.23	1.14–1.32	<0.001	1.28	1.16–1.40	<0.001	-	-	-
Vomiting = Yes	1.37	1.22–1.54	<0.001	1.31	1.12–1.52	<0.001	-	-	-
Abdominal pain = Yes	1.24	1.07–1.42	0.004	0.91	0.76–1.10	0.349	-	-	-
Skin bleeding = Yes	4.82	3.87–5.99	<0.001	2.08	1.53–2.84	<0.001	-	-	-
Mucosal bleeding = Yes	2.83	2.15–3.73	<0.001	1.02	0.67–1.53	0.934	-	-	-
Flush = Yes	2.02	1.75–2.34	<0.001	1.37	1.11–1.69	0.003	-	-	-
Hepatomegaly = Yes	2.85	0.87–9.36	0.086	0.58	0.05–7.08	0.687	-	-	-
Rash = Yes	2.02	1.75–2.34	<0.001	1.23	0.78–1.93	0.381	-	-	-
Injection = Yes	2.40	2.05–2.81	<0.001	1.58	1.25–1.99	<0.001	-	-	-
**Laboratory results**									
WBC (+103/mm3)	0.70	0.68–0.71	<0.001	0.77	0.75–0.80	<0.001	0.78	0.76–0.80	<0.001
PLT (+104/mm3)	0.87	0.86–0.88	<0.001	0.97	0.95–0.98	<0.001	0.94	0.93–0.95	<0.001
HCT (+1%)	1.12	1.10–1.14	<0.001	0.97	0.95–0.99	0.013	-	-	-
ALB (+1g/l)	0.97	0.95–0.99	0.004	1.00	0.98–1.03	0.787	-	-	-
AST (per 2-fold increase)	3.65	3.23–4.14	<0.001	3.50	2.97–4.13	<0.001	-	-	-
CK (per 2-fold increase)	1.33	1.24–1.43	<0.001	0.84	0.76–0.93	<0.001	-	-	-

BMI: body mass index; WBC: white blood cell count; PLT: platelet count; HCT: hematocrit; ALB: albumin; AST: aspartate aminotransferase; CK: creatine kinase

In settings where NS1 rapid tests are routinely used, the EDC can be combined with the NS1 rapid test as a composite test (classified as positive when either NS1 rapid test or EDC are positive, and classified as negative when both NS1 rapid test and EDC are negative). This composite test had sensitivity of 91.6% (95%CI: 90.4–92.9%) while the specificity was 75.7% (95%CI: 74.5–77.0%). Corresponding positive and negative predictive values were 61.7% (95%CI: 60.6–63.1%) and 95.5% (95%CI: 94.9–96.1%). If a higher specificity was desired, a higher cut-off value of the EDC could be used for the combined test instead, e.g. a cut-off of 50% would lead to a sensitivity of 86.0% (95%CI: 84.5–87.6%) and specificity of 89.6% (95%CI: 88.7–90.5%). These results imply that the EDC is useful in settings with and without NS1 rapid testing.

### User-friendly applications of the Early Dengue Classifier

**Fig 2:**
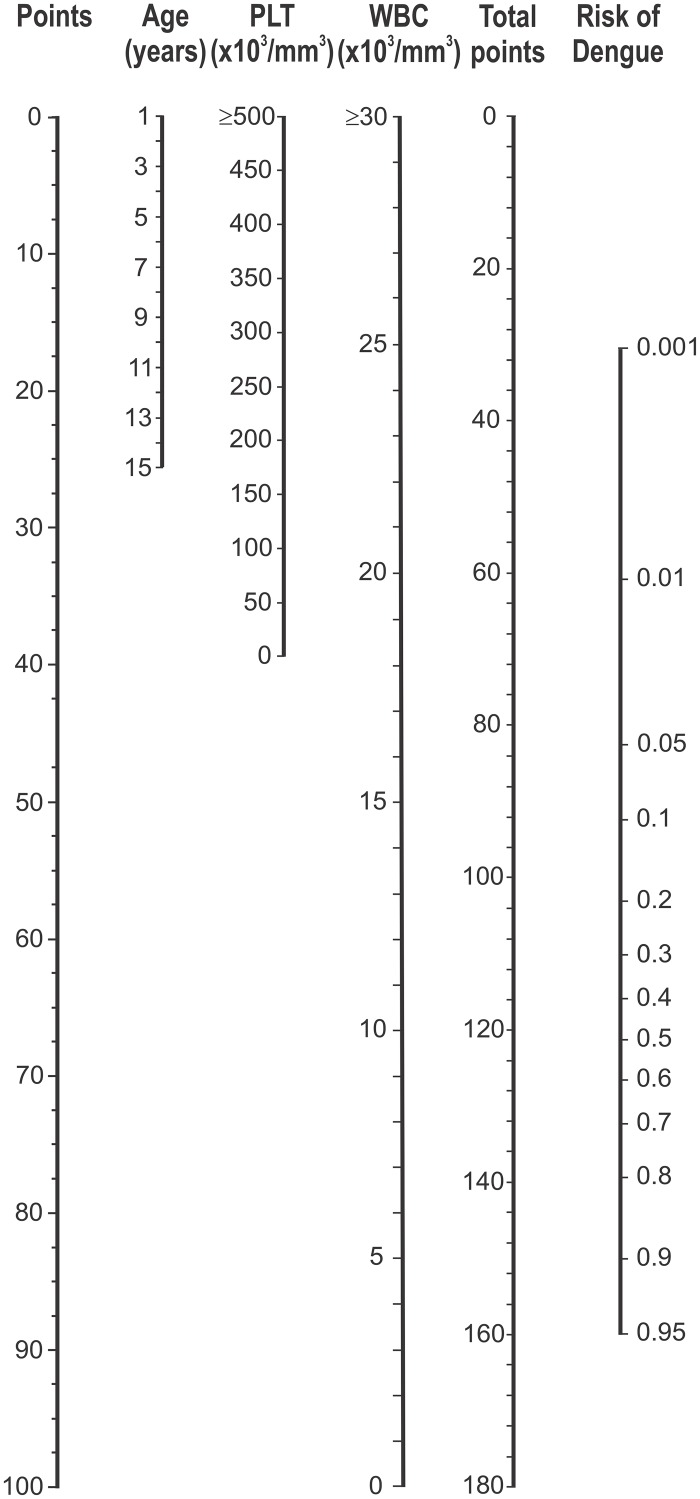
Nomogram of the Early Dengue Classifier (EDC) to predict the risk of dengue. A horizontal line from a predictor value to the “Points” axis assigns points to the 3 required variables age, platelet count (PLT), and white blood cell count (WBC). The sum of these points (total points) can then be translated to the corresponding predicted risk of dengue. As an example, a 9-year-old patient with a PLT of 100x10^3^/mm^3^, and a WBC of 5x10^3^/mm^3^ has a score of 15+32+84 = 131, and the corresponding risk of dengue is about 70%. Note: As <1% of patients had platelet (PLT) count >500x10^3^/mm^3^ or white blood cell (WBC) count >30x10^3^/mm^3^, for better visualization, PLT and WBC counts were truncated at 500x10^3^/mm^3^ and 30x10^3^/mm^3^ respectively.

Fig 2 presents a nomogram of the EDC. The nomogram assigns points to all risk factors and translates the total point score to a predicted risk for dengue. For example, a 9-year-old patient with platelet count 100x10^3^/mm^3^, and white blood cell count 5x10^3^/mm^3^ has a total points score of 15+32+84 = 131, and the corresponding risk of dengue is about 70%. The predicted risk of dengue is larger than 33.3% so the patient would be classified as dengue positive. Alternatively, the EDC could be implemented as a smartphone app. The exact formula for the estimated risk of dengue (p) is given by the following logistic equation: logit(p) = 1.236 + 0.139*age (in years)– 0.254*white blood cell (in 10^3^/mm^3^)– 0.006 *platelet (in 10^3^/mm^3^).

## Discussion

The early and accurate diagnosis of dengue on the grounds of clinical signs and symptoms alone is problematic [9]. Physicians need better tools to assist in early diagnosis if the WHO ambition of a 50% reduction in global dengue mortality is to be achieved by 2020. This study characterized the performance of three diagnostic approaches; the NS1 rapid test, a stand-alone diagnostic classifier and the combination of NS1 rapid test and diagnostic classifier together. Our results highlight the utility of NS1 rapid tests for an early specific diagnosis, yet also remind that 2^nd^ generation tests are needed with improved sensitivity. The diagnostic classifier described here could help guide diagnosis in endemic settings, or be used as an adjunct to help exclude dengue in patients returning a negative NS1 rapid test result.

There is a body of literature describing the performance of NS1 rapid tests for the diagnosis of dengue [6–8, 19–22]. This current study extends that literature in several ways. First, by virtue of the large sample size we demonstrate with high precision the differential sensitivity of the NS1 Ag STRIP for different DENV serotypes. This test was sensitive (between 75–85%) for DENV-1, -3 and -4 infections, but poorly sensitive in DENV-2 infections (46.4%). Lower sensitivity was partially attributable to the great majority of DENV-2 infections being associated with secondary serological responses, although we note sensitivity was also low in primary DENV-2 infections. This suggests that there are particular virological (e.g. lower viral burdens in vivo) or intrinsic aspects of the NS1 test, that limit DENV-2 NS1 detection. [23–26]. Second, we make the novel observation that 5–10 fold enrichment of proteins with molecular weight >100kDa in plasma specimens (the NS1 hexamer has predicted molecular weight of 310kDa [27]) did not lead to improved NS1 detection rates. These data suggest that dengue patients who return negative NS1 rapid test results in the first 3 days of illness have free plasma NS1 concentrations substantially below the limit of sensitivity of existing assays and that 2^nd^ generation tests might need to be at least an order of magnitude more sensitive. Nonetheless, better NS1 rapid diagnostic tests are needed if they are going to be widely adopted by clinical services in primary care settings. In malaria, HRP2 rapid diagnostic tests for *Plasmodium falciparum* infection are an example of how improvements to assay performance can lead to recognition as a diagnostic standard of care [28]. Finally, although serum NS1 concentrations have been proposed to have prognostic value in a small study, this is yet to be independently validated and is likely to be difficult given that blood NS1 concentrations vary widely according to the infecting DENV serotype, serological response and day of illness [8, 24, 29, 30].

Previous studies have described clinical and/or routine laboratory findings that distinguish patients with dengue from those with other febrile illnesses [12, 31–37]. What is striking in the literature is that only three prospective studies have considered dengue diagnostic algorithms exclusively in children and of these the largest contained 1227 patients, of who 614 had dengue [11, 38, 39]. More generally, most diagnostic studies failed to report positive and negative predictive values for their diagnostic algorithms, thus making it difficult to assess their utility in routine practice. Against this backdrop, a strength of the current study is the large sample size, the presence of all four DENV serotypes, robust statistical validation techniques and transparent performance characteristics. The clinical signs and symptoms that make up the WHO case definition for dengue were not used in the final, parsimonious diagnostic EDC classifier. Instead, we found that only three variables—patient age, white blood cell count and platelet count, provided similar discriminatory information as alternative models that relied upon a much larger set of clinical data.

The purpose of this study was to explore whether it was possible to develop any kind of simple, evidence-based algorithm for early diagnosis—the results demonstrate this feasibility, albeit the performance characteristics of the end-result algorithm are not so outstanding that they will result in widespread adoption or change the practice of experienced clinicians. We concur with Potts *et al* in the belief that diagnostic rules for dengue are not a replacement for good clinical acumen and management [9]. Nonetheless, the EDC described here offers an evidence-based guide that can likely improve the prevailing diagnostic accuracy of most Vietnamese physicians working in primary care who do not possess extensive experience in dengue diagnosis and management. In particular, in settings where NS1 rapid tests are not routinely available or affordable, or where DENV-2 is the most prevalent virus in circulation, the EDC could help guide clinicians in making their differential diagnosis. An early diagnosis of dengue can assist in patient triage and management by directing clinical/caregiver attention to clinical warning signs and/or the appearance of capillary permeability, for which supportive oral and/or parenteral fluid therapy is recommended in order to prevent circulatory compromise. Additionally, in the first days of illness many dengue cases are infectious to *Aedes aegypti* mosquitoes and hence an early diagnosis could support measures to prevent further transmission, e.g. by use of topical repellents and local mosquito control [40].

Our study has several design features and limitations that might preclude its wider generalizability. The EDC relies on routine hematology findings that are commonly accessible in primary care settings in Vietnam but might not be available everywhere. By design, our study focused on patients with <72 hours of illness and hence our results might not be applicable to patients who present to medical care at later time-points. By using the age of the patient as a component of the EDC, it’s likely that the EDC would not perform well in settings where the burden of dengue falls on age-groups different from that in southern Vietnam. Nonetheless, this study has delivered the largest population-based and quantitative framework to guide early diagnosis of pediatric dengue. Further prospective validation in Vietnam and other endemic countries with similar epidemiology will be needed to establish the clinical utility of the EDC.

## Supporting Information

S1 Fig STARD flow chart showing patient enrolment and classification. (DOC)

S2 Fig Plots of estimated component smooth functions of a GAM for the risk of dengue which included all candidate predictors listed in S1 Table and modeled continuous parameters as smooth terms. Only terms estimated to have a non-linear association with outcome are displayed. Dots correspond to individual partial residuals; solid lines correspond to smooth spline functions estimated by GAM; dashed lines correspond to the estimated smooth functions plus/minus one standard error.
(DOCX)

S1 Table Clinical and demographic features recorded at the time of study enrolment. (DOCX)

S2 Table Sensitivity of NS1 rapid test according to serotype and serological response in hospitalized patients. (DOCX)

S3 Table Detection of NS1 in viremic blood samples collected at the time of enrolment, before and after volume concentration. (DOCX)

S4 Table Performance of models for the risk of dengue in all subjects resulting from different statistical modeling approaches. (DOCX)

S5 Table Performance of the Early Dengue Classifier (EDC). (DOCX)

S1 Text Detailed statistical appendix including assessment of linearity and interactions. (DOCX)

S1 Dataset Early Dengue classifier dataset. Clinical and laboratory results (line listings) by individual patient, plus data dictionary.
(XLS)

S1 Checklist STARD checklist. (DOC)
