# Supplementary figures and images for: Sensitivity and Specificity of a Novel Classifier for the Early Diagnosis of Dengue
Source: PLoS Negl Trop Dis. 2015 Apr 2;9(4):e0003638. doi: 10.1371/journal.pntd.0003638 (PMC4383489; doi:10.1371/journal.pntd.0003638)

**
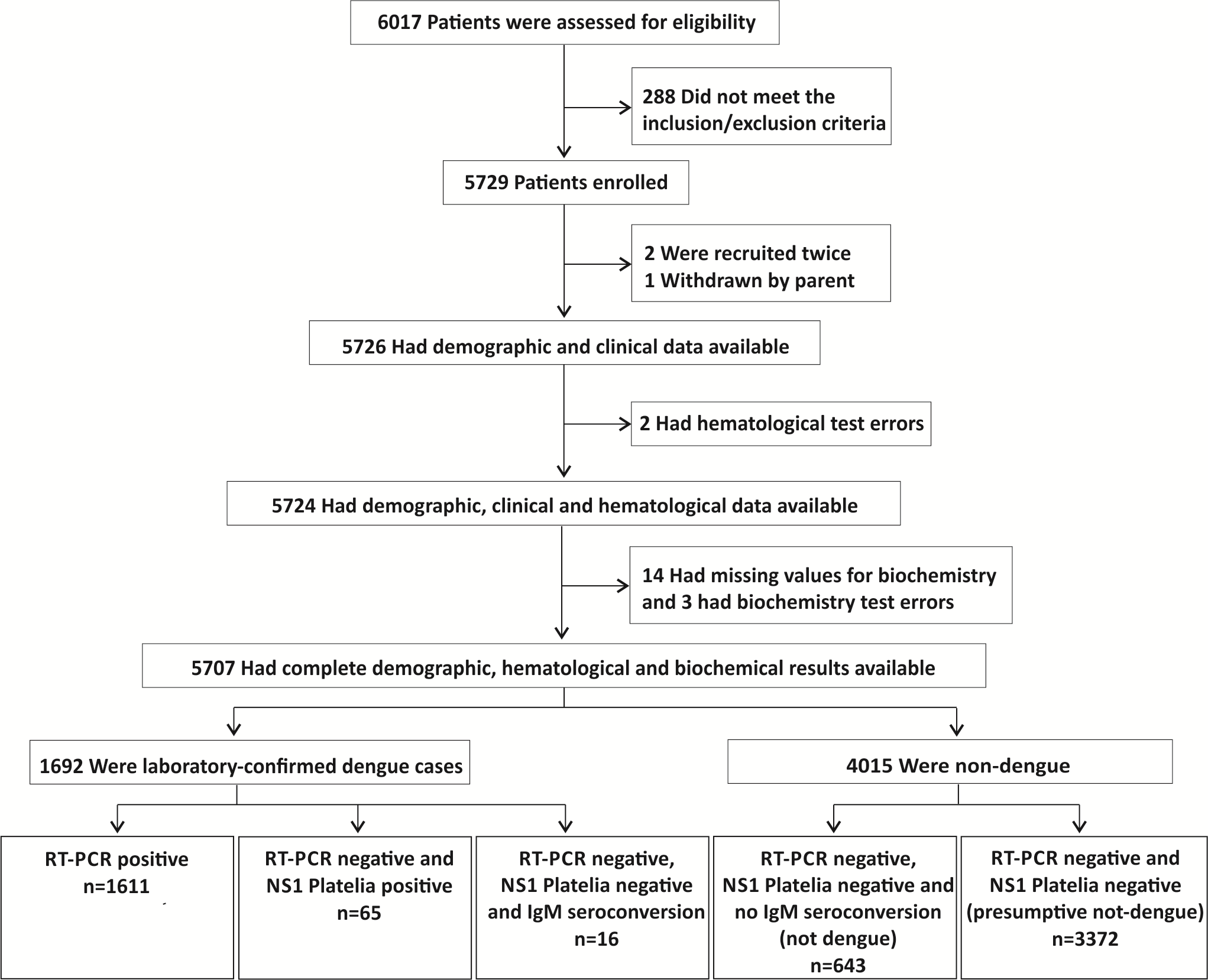
**

**S1 Fig.** Flow chart showing patient enrolment and classification.

Supplement: S1 Fig — (DOC) [file pntd.0003638.s001.doc]
