# Supplementary material for: Sensitivity and Specificity of a Novel Classifier for the Early Diagnosis of Dengue
Source: PLoS Negl Trop Dis. 2015 Apr 2;9(4):e0003638. doi: 10.1371/journal.pntd.0003638 (PMC4383489; doi:10.1371/journal.pntd.0003638)
Supplement: S2 Fig — Only terms estimated to have a non-linear association with outcome are displayed. Dots correspond to individual partial residuals; solid lines correspond to smooth spline functions estimated by GAM; dashed lines correspond to the estimated smooth functions plus/minus one standard error. (DOCX) [file pntd.0003638.s002.docx]

**
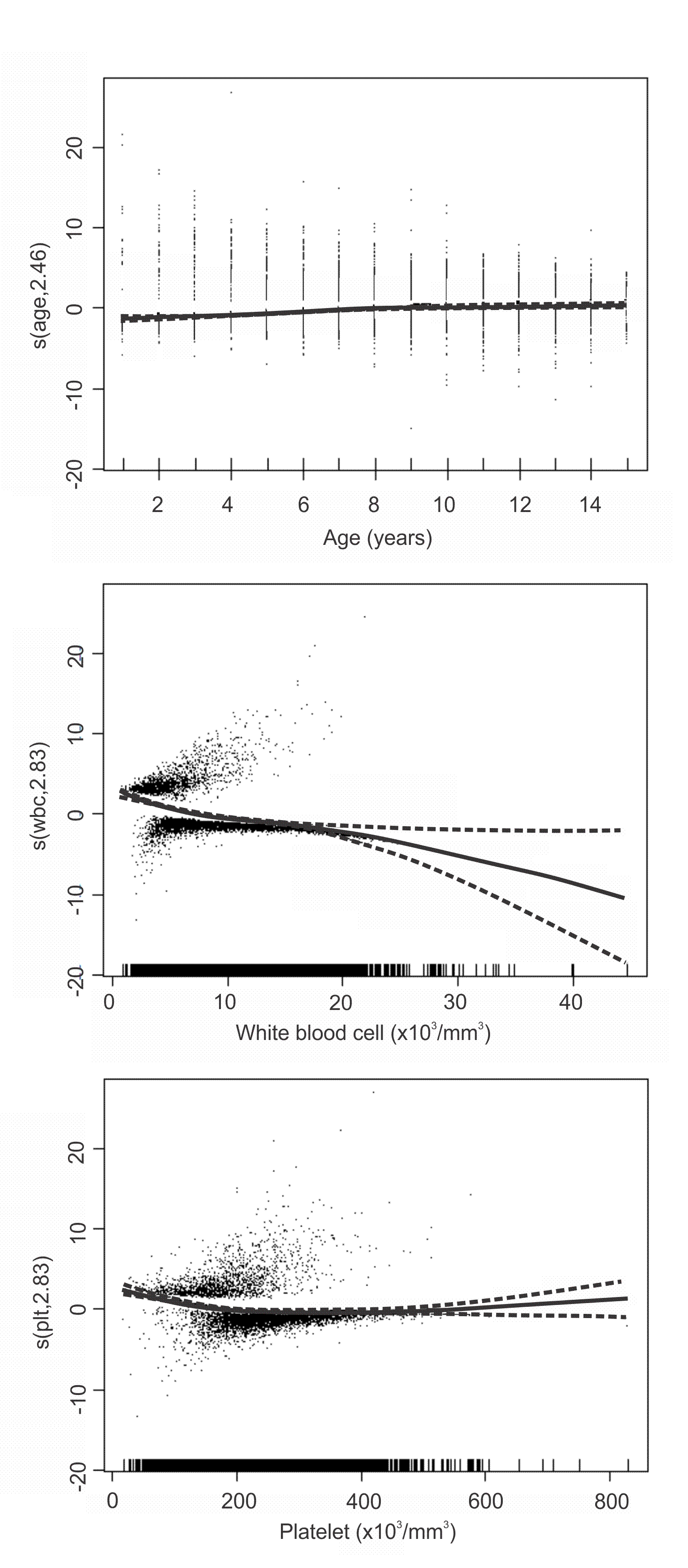
**

**S2 Fig.** Plots of estimated component smooth functions of a GAM for the risk of dengue which included all candidate predictors listed in Supplementary Table 1 and modeled continuous parameters as smooth terms. Only terms estimated to have a non-linear association with outcome are displayed. Dots correspond to individual partial residuals; solid lines correspond to smooth spline functions estimated by GAM; dashed lines correspond to the estimated smooth functions plus/minus one standard error.
