# Supplementary material for: Sensitivity and Specificity of a Novel Classifier for the Early Diagnosis of Dengue
Source: PLoS Negl Trop Dis. 2015 Apr 2;9(4):e0003638. doi: 10.1371/journal.pntd.0003638 (PMC4383489; doi:10.1371/journal.pntd.0003638)
Supplement: S1 Table — (DOCX) [file pntd.0003638.s003.docx]

**S1 Table.** **Clinical and demographic features recorded at the time of study enrolment**

| Variable | Definition |
| --- | --- |
| 1. Day of illness (days) | Day of illness at enrollment |
| 1. Age (years) | Age in years |
| 1. Sex | Male/Female |
| 1. BMI (kg/(m)^2^) | Body mass index (BMI) = weight (kg) /height (m) ^2 |
| 1. Temperature at enrolment (°C) | Axillary temperature at enrollment |
| 1. Vomiting (Yes/No) | Any episode of vomiting in history of illness |
| 1. Skin bleeding (Yes/No) | History or clinical examination shows that the patient has skin bleeding, e.g. petechiae or ecchymosis. |
| 1. Mucosal bleeding (Yes/No) | Any frank bleeding from any mucosal site e.g. epistaxis, gingival bleeding, gastrointestinal bleeding, or urogenital bleeding in history of illness or medical examination |
| 1. Abdominal pain (Yes/No) | History or clinical examination shows that the patient has a painful feeling at the abdomen by self-reporting or elicited on gentle palpation. |
| 1. Rash (Yes/No) | Clinical examination reveals generalised macular, blanching, non-petechial rash. |
| 1. Flush (Yes/No) | Redness of the skin caused by dilatation and congestion of the capillaries that blanches with pressure. |
| 1. Conjunctival injection (Yes/No) | Redness (bright red or pink) of the conjunctiva fading towards the limbus due to dilatation of the superficial conjunctival blood vessels. |
| 1. WBC (10^3^/mm^3^) | Absolute white blood cell count at enrollment |
| 1. HCT (%) | Haematocrit at enrollment |
| 1. Platelet count (10^3^/mm^3^) | Platelet count at enrollment |
| 1. ALB (g/L) | Blood albumin concentrations |
| 1. AST (U/l) | Blood aspartat aminotransferase concentrations (log2-transformed) |
| 1. CK (U/l) | Blood creatine kinase concentrations (log2-transformed) |
