# Supplementary material for: Sensitivity and Specificity of a Novel Classifier for the Early Diagnosis of Dengue
Source: PLoS Negl Trop Dis. 2015 Apr 2;9(4):e0003638. doi: 10.1371/journal.pntd.0003638 (PMC4383489; doi:10.1371/journal.pntd.0003638)
Supplement: S2 Table — (DOCX) [file pntd.0003638.s004.docx]

**S2 Table: Sensitivity of NS1 rapid test according to serotype and serological response in hospitalized patients**

|  | **Sensitivity % (95%CI)** | |
| --- | --- | --- |
|  | Primary infection  (NS1+ /total primary infection)^a^ | Secondary infection  (NS1+ /total secondary infection)^b^ |
| DENV-1 | 93.6% (87.2-97.4%)  (107/109) | 82.2% (75.5-87.8%)  (134/163) |
| DENV-2 | 42.9% (17.8-71.1%)  (6/14) | 57.1% (48.9-64.9%)  (89/156) |
| DENV-3 | 95.2% (76.1-99.2%)  (20/21) | 81.1% (64.8-92.0%)  (30/37) |
| DENV-4 | 70.0% (45.7-88.0%)  (14/20) | 81.1% (74.8-86.4%)  (154/190) |
| Unknown | 68.8% (41.4-88.9%)  (11/16) | 55.5% (30.8-78.4%)  (10/18) |
| Total | **180** | **564** |
| *Note: (a), (b) indicated the number of cases with NS1 rapid test positive compared to the total number of primary or secondary infections respectively within the same individual dengue serotypes.* | | |
