# Supplementary material for: Sensitivity and Specificity of a Novel Classifier for the Early Diagnosis of Dengue
Source: PLoS Negl Trop Dis. 2015 Apr 2;9(4):e0003638. doi: 10.1371/journal.pntd.0003638 (PMC4383489; doi:10.1371/journal.pntd.0003638)
Supplement: S3 Table — (DOCX) [file pntd.0003638.s005.docx]

**S3 Table. Detection of NS1 in viremic blood samples collected at the time of enrolment, before and after volume concentration**

|  |  |  |  |  |  |  |  |
| --- | --- | --- | --- | --- | --- | --- | --- |
|  |  |  |  | **NS1 status (Platelia ELISA)^a^** | | |  |
| **Sample#** | **DENV Serotype** | **log10 viremia RNA copies/ml** | **NS1 rapid test** | **Original sample** | **Sample concentrate** | **Sample filtrate** | **Fold concentration of sample** |
| 15-222 | 2 | 6.85 | - | + | + | - | 8.0 |
| 16-427 | 2 | 6.90 | - | + | + | - | 6.7 |
| 16-134 | 1 | 9.54 | - | + | + | + | 6.7 |
| 16-139 | 1 | 6.93 | - | + | + | + | 6.7 |
| 16-142 | 1 | 8.22 | - | + | + | + | 8.0 |
| 16-130 | 1 | 8.34 | - | + | + | - | 6.7 |
| 1-920 | 1 | 8.33 | - | + | + | + | 8.0 |
| 16-184 | 1 | 7.95 | - | + | + | - | 8.0 |
| 16-223 | 2 | 6.33 | - | + | + | - | 10.0 |
| 16-269 | 1 | 5.40 | - | - | - | - | 6.7 |
| 16-281 | 2 | 4.98 | - | - | - | - | 6.7 |
| 16-308 | 1 | 5.19 | - | - | - | - | 5.7 |
| 16-352 | 2 | 5.39 | - | - | + | - | 5.7 |
| 16-399 | 2 | 4.49 | - | - | - | - | 4.0 |
| 15-226 | 2 | 8.47 | - | - | - | - | 5.7 |
| 16-155 | 1 | 3.11 | - | - | - | - | 5.7 |
| 16-160 | 1 | 7.56 | - | - | - | - | 5.7 |
| 16-514 | 1 | 7.36 | - | Eq | Eq | - | 5.7 |
| 16-154 | 1 | 2.94 | - | - | - | - | 6.7 |
| 1-846 | 2 | 5.78 | - | - | - | - | 5.7 |
| 1-882 | 1 | 11.05 | - | - | - | - | 8.0 |
| ^a^ NS1 Platelia test result: "+"=positive, "-"=negative, “Eq’= equivocal | | | | | | | |
