# Supplementary material for: Sensitivity and Specificity of a Novel Classifier for the Early Diagnosis of Dengue
Source: PLoS Negl Trop Dis. 2015 Apr 2;9(4):e0003638. doi: 10.1371/journal.pntd.0003638 (PMC4383489; doi:10.1371/journal.pntd.0003638)
Supplement: S4 Table — (DOCX) [file pntd.0003638.s006.docx]

**S4 Table: Performance of models for the risk of dengue in all subjects resulting from different statistical modeling approaches**

|  | Full model | Step-wise AIC | STAB | CART | RF |
| --- | --- | --- | --- | --- | --- |
| Calibration intercept | -0.414 (-0.470–0.554) | -0.432 (-0.470–0.557) | -0.200 (-0.810–0.852) | -0.211 (-0.667–0.833) | -0.256 (-0.601–0.742) |
| Calibration slope | 0.926 (0.820–1.248) | 0.920 (0.820–1.263) | 1.075 (0.855–1.272) | 1.076 (0.829–1.076) | 1.293 (1.037–1.331) |
| AUC | 0.855 (0.815–0.861) | 0.854 (0.815–0.861) | 0.835 (0.792–0.850) | 0.774 (0.703–0.777) | 0.872 (0.830–0.873) |
| Sensitivity (cutoff 0.33) | 0.785 (0.651–0.868) | 0.790 (0.659–0.890) | 0.795 (0.659–0.912) | 0.614 (0.459–0.736) | 0.812 (0.579–0.846) |
| Specificity (cutoff 0.33) | 0.760 (0.699–0.868) | 0.756 (0.699–0.868) | 0.726 (0.550–0.839) | 0.896 (0.735–0.939) | 0.809 (0.709–0.873) |
| PPV (cutoff 0.33) | 0.535 (0.513–0.699) | 0.533 (0.519–0.720) | 0.505 (0.426–0.738) | 0.676 (0.504–0.848) | 0.600 (0.513–0.768) |
| NPV (cutoff 0.33) | 0.909 (0.815–0.936) | 0.911 (0.811–0.946) | 0.909 (0.748–0.945) | 0.868 (0.702–0.884) | 0.924 (0.805–0.926) |
| The full model included all variables listed in supplementary table 1 plus interaction terms between age and WBC and between age and PLT.  Step-wise AIC selected the following variables: age, day of illness, BMI, vomiting, temperature, skin bleeding, flush, injection, hematocrite, log2(aspartate transaminase), log2(creatinine kinase), white blood cell count, platelet count, age by white blood cell count and age by platelet count.  STAB selected the following variables from the full model: age, white blood cell count, platelet count.  CART selected the following variables: age, white blood cell count.  RF was derived from all variables listed in supplementary table 1.  AUC: area under the ROC curve, PPV: positive predictive value, NPV: negative predictive value, Step-wise AIC: step-wise backwards variable selection using Akaike information criterion, STAB: stability selection, CART: classification and regression trees, RF: random forests.  The results were from leave-one-site-out validation and were presented by mean and range of performances across left out sites. | | | | | |
