# Supplementary material for: Sensitivity and Specificity of a Novel Classifier for the Early Diagnosis of Dengue
Source: PLoS Negl Trop Dis. 2015 Apr 2;9(4):e0003638. doi: 10.1371/journal.pntd.0003638 (PMC4383489; doi:10.1371/journal.pntd.0003638)
Supplement: S5 Table — (DOCX) [file pntd.0003638.s007.docx]

**S5 Table. Performance of the Early Dengue Classifier (EDC)**

|  | All patients | Dry season | Wet season | Temporal validation | Leave-one-site-out validation |
| --- | --- | --- | --- | --- | --- |
| Calibration intercept | 0 | 0 | 0 | -0.247 | -0.200  (-0.810–0.852) |
| Calibration slope | 1 | 1 | 1 | 0.940 | 1.075  (0.855–1.272) |
| AUC | 0.829 | 0.831 | 0.825 | 0.776 | 0.835  (0.792–0.850) |
| Sensitivity (cutoff 0.33) | 0.748  (0.730–0.768) | 0.731  (0.726–0.750) | 0.783  (0.771–0.798) | 0.786  (0.770–0.812) | 0.795  (0.659–0.912) |
| Specificity (cutoff 0.33) | 0.763  (0.752–0.776) | 0.792  (0.781–0.803) | 0.758  (0.742–0.771) | 0.595  (0.583–0.612) | 0.726  (0.550–0.839) |
| PPV (cutoff 0.33) | 0.571  (0.562–0.590) | 0.587  (0.579–0.602) | 0.570  (0.561–0.586) | 0.460  (0.451–0.482) | 0.505  (0.426–0.738) |
| NPV (cutoff 0.33) | 0.878  (0.868–0.885) | 0.911  (0.889–0.925) | 0.864  (0.851–0.972) | 0.863  (0.850–0.877) | 0.909  (0.748–0.945) |
| Table shows apparent performance (95%CI) in all patients and seasons; performance (95%CI) in the validation set for temporal validation; and mean (range) of performances across left out sites for leave-one-site-out validation. Dry season is January to June, and wet season is July to December. The proportion of true dengue cases in all patients, dry season and wet season was 29.6% (1692/5707), 21.1 % (405/1916) and 33.9% (1287/3791) respectively. | | | | | |
