# Supplementary material for: Sensitivity and Specificity of a Novel Classifier for the Early Diagnosis of Dengue
Source: PLoS Negl Trop Dis. 2015 Apr 2;9(4):e0003638. doi: 10.1371/journal.pntd.0003638 (PMC4383489; doi:10.1371/journal.pntd.0003638)
Supplement: S1 Text — (DOCX) [file pntd.0003638.s008.docx]

**S1 text**

*Assessment of linearity*

A generalized additive model (GAM) with integrated smoothness estimation as implemented in the R package mgcv version 1.8-0 was used to assess potential non-linear effects of variables on the risk of dengue. The model included all candidate predictors listed in Supplementary Table 1 and modeled continuous parameters as smooth terms.

The GAM chose non-linear associations for several parameters including age, white blood cell count, and platelet count (Supplementary Figure 2). While the estimated association of outcome with age and white plot cell count was not perfectly linear, it was monotone and reasonably close to a linear function. Hence, these variables were treated as linear terms.

The estimated effect of platelet count on the risk of dengue in Supplementary Figure 2 indicates a decreasing risk of dengue for increasing platelet counts up to ~300x10^3^/mm^3^ but a slight increase in the risk of dengue for higher platelet counts. However, the performance of the final model in which platelet count was modeled non-linearly with a linear spline function with one knot at 300x10^3^/mm^3^ was similar to the one in which platelet was included as a linear term (AUC= 0.83 vs. AUC=0.829). In addition, it seemed unclear whether it could be physiopathologically plausible for dengue patients to have abnormally high platelet counts or whether this was just an artifact. Thus, we presented in the main paper the results of the models in which platelet count was treated as linear term.

*Assessment of interactions*

Potential interactions between age, sex, study site and hematological and biochemical results were examined by likelihood ratio tests based on the full model with all variables from Supplementary Table 1.

Interaction terms between age and white blood cell count (p<0.001) and between age and platelet count (p=0.039) were significant and added to the full starting model.
